# A machine learning approach to predict healthcare cost of breast cancer patients

**DOI:** 10.1038/s41598-021-91580-x

**Published:** 2021-06-14

**Authors:** Pratyusha Rakshit, Onintze Zaballa, Aritz Pérez, Elisa Gómez-Inhiesto, Maria T. Acaiturri-Ayesta, Jose A. Lozano

**Affiliations:** 1 Basque Center for Applied Mathematics, Bilbao, Spain; 2 Osakidetza, Bilbao, Spain

**Keywords:** Computer science, Health care, Health care economics

## Abstract

This paper presents a novel machine learning approach to perform an early prediction of the healthcare cost of breast cancer patients. The learning phase of our prediction method considers the following two steps: (1) in the first step, the patients are clustered taking into account the sequences of actions undergoing similar clinical activities and ensuring similar healthcare costs, and (2) a Markov chain is then learned for each group to describe the action-sequences of the patients in the cluster. A two step procedure is undertaken in the prediction phase: (1) first, the healthcare cost of a new patient’s treatment is estimated based on the average healthcare cost of its *k*-nearest neighbors in each group, and (2) finally, an aggregate measure of the healthcare cost estimated by each group is used as the final predicted cost. Experiments undertaken reveal a mean absolute percentage error as small as 6%, even when half of the clinical records of a patient is available, substantiating the early prediction capability of the proposed method. Comparative analysis substantiates the superiority of the proposed algorithm over the state-of-the-art techniques.

## Introduction

An *electronic health record* (EHR) is an electronic version of a patient’s clinical history over time. It comprises all administrative clinical data of a patient in a healthcare organization, including his/her demographics, diagnosis, medications, laboratory data, and associated costs, and so on. The plethora of longitudinal patients’ data of an EHR can be utilized for developing patient-centered personalized healthcare solutions, including cost. It is however worth mentioning that the healthcare costs, ranging from clinician’s fees to the cost of hospital stays and medicines, are escalating at a rapid rate around the world^1, 2^. It has motivated the researchers to take keen interest in controlling this upsurge in the healthcare costs. The crucial step to control the healthcare cost is to enable the healthcare organizations to predict the possible future cost of individual patients. It in turn helps to identify the individuals at the highest risk of enduring the significant costs in future. It thus helps to prioritize the allocation of scarce resources among the patients in a healthcare organization for efficient care management.

Moreover, a report from The Commonwealth Fund (2012) emphasizes the need to identify high-cost patients as the first step towards achieving “rapid improvements in the value of services provided”^3^. A proactive approach to address this problem is to identify patients who are at risk of becoming high-cost patients accurately before substantial unnecessary costs have been incurred and health condition has deteriorated further. Eventually, this calls for prediction of possible total healthcare cost of a patient as early as possible when a limited volume of clinical records of the given patient is provided. In other words, another important aspect in the context of healthcare cost prediction is to devise a model using a training set of complete clinical records of some patients to predict the total healthcare cost of a new patient as accurately and also as early as possible, preferably before the availability of the patient’s full-length clinical record. Such early prediction of future healthcare cost can be used to judiciously identify high-risk high-cost patients and prevent crises in healthcare organizations. It is obvious that the earliness of the prediction may affect the accuracy. It has motivated the researchers to build a model to predict healthcare cost as early as possible while maintaining an appropriate level of accuracy.

Nevertheless, healthcare cost prediction based on individual patient’s characteristics is a challenging issue from the data mining perspective due to the non-Gaussian skewed distribution of the cost data of the patients^4^. Studies in^5, 6^ reveal dubious efficacy of the statistical methods to predict the healthcare cost. Furthermore, the traces of linear regression and rule-based approaches are also found in literature^2, 6^ for the cost prediction. But the requirement of a lot of domain knowledge has restricted their applications for most of the real world economic data of the patients^7^. Now-a-days, machine learning algorithms, including clustering and classification techniques, have emerged as an alternative effective tool for this purpose^8, 9^.

This paper proposes a machine learning based novel approach for healthcare cost prediction of individual patient’s treatments based on their clinical actions, jointly including the clinical activities and the respective cost over time. The activity here represents diagnosis, medication, pharmacy and the like. A two-step procedure is employed in the learning phase: (1) in the first step, the ordered sequences of clinical actions of the patients’ treatments are clustered using the hierarchical DBSCAN^10^ with an aim to identify the group of patients undertaking similar clinical activities and incurring similar healthcare costs, and (2) each group is then modelled by means of a Markov chain^11^ delineating the probability distributions of transitions between different clinical actions. A new distance measure is also proposed to measure the similarity of the treatment patterns of the patients during clustering.

The prediction phase, concerned with prediction of the healthcare cost of the sequence of clinical actions of a new patient’s treatment, also encompasses two steps: (1) first, for each group, we compute a tentative cost of the new sequence by averaging the cost of its *k*-nearest neighbor^12^ sequences in the group, (2) the final cost is obtained as a weighted sum of the cost estimated by each of the groups. The weights for each group are the likelihood of the new sequence to the respective group as assigned by the corresponding Markov chain.

The performance of the proposed healthcare cost prediction algorithm is evaluated with the economic information together with information of the clinical activities of the breast cancer patients obtained from the health administrative department of the public health care system of the Basque Country, Spain. A 10-fold cross validation is employed with the training dataset resulting the optimal value of *k* of *k*-NN as three in the present application with respect to the mean absolute percentage error (*MAPE*)^2^. Moreover, the proposed method results in an *MAPE* measure of less than 6% when half of the clinical records of a new patient is available, irrespective of the value of *k*. It substantiates the capability of the proposed stratagem for early prediction of healthcare cost. Experiments undertaken also reveal that the proposed algorithm outperforms its state-of-the-art contenders with respect to *MAPE* metric. The comparative analyses verify the significance of jointly considering the clinical activity and the associated cost data to effectively capture the clinical records of patients for accurate healthcare cost prediction as early as possible.

The paper is divided into following sections. Second section delineates the proposed method of healthcare cost prediction. Experiments undertaken and the results are reported in third section. Fourth section concludes the paper.

## Method

### Data transformation

This section refers to transforming the database of individual patient’s treatments into a series of actions, sorted by time. Here, we provide some definitions which will be used throughout the paper to develop a solution to the healthcare cost prediction problem.

#### Definition-1: Action

Let **X** be the set of all clinical activities, including diagnosis, procedure, medicine and the like, ${{\mathbf{Y}}} \in \mathbb {R}$ be the set of all possible incurred healthcare cost as recorded in the database and **T** be the set of visiting times of the patients to the hospital. An *action*, say *a*, is then expressed as a three-tuple, given by

1 $$\begin{aligned} a = \{ (\ x, y, t )\ | \forall x \in {\mathbf{X}} , \forall y \in {\mathbf{Y}} , \forall t \in {\mathbf{T}} \}. \end{aligned}$$

#### Definition-2: Patient’s treatment

A *patient’s treatment* is defined by a sequence of its corresponding actions, sorted by the visiting time. Symbolically, a patient’s treatment *P* is represented by

2 $$\begin{aligned} P = (a_1, a_2, \ldots , a_n) \end{aligned}$$

where $a_i = (x_i, y_i, t_i)$ represents the action encompassing the clinical activity $x_i\in {\mathbf{X}}$ and its respective healthcare cost $y_i\in {\mathbf{Y}}$ incurred during visiting time $t_i\in {\mathbf{T}}$ of the specific patient. For sake of simplicity of readers, we drop the notion of visiting time and hence $a_i$ now can be simplified as

3 $$\begin{aligned} a_i = \{(\ x_i, y_i)\ | x \in {\mathbf{X}} , y \in {\mathbf{Y}} \}. \end{aligned}$$

The clinical actions of *P* in (2) are chronologically ordered. Evidently, if $i < j$, $a_i$ occurs before $a_j$. A sequence of actions of a patient’s treatment is used to jointly track the progression of its activity-outcome and the corresponding healthcare cost over time. The length of the sequence varies across patients because of the diversity in their treatments over time.

#### Definition-3: Modified cost

Intuitively, the number of possible actions for all patients in the database is huge due to infinite number of healthcare cost elements in **Y**. For the sake of simplicity, **Y** is reduced to a finite set in a two step procedure described below.

1. *Discretization*: First, the entire range of **Y** is discretized into $n_s$ segments defined by the $n_s$-quantiles of **Y**. In other words, we set the lower and the upper limit of the *i*-th segment respectively to the $(i-1)$-th quantile and the *i*-th quantile of the healthcare cost elements for all possible clinical activities, recorded in the database.
2. *Quantization*: Then a real healthcare cost element, lying in the *i*-th segment is replaced by the mean value of all cost elements of the *i*-th segment.

**Figure 1:**
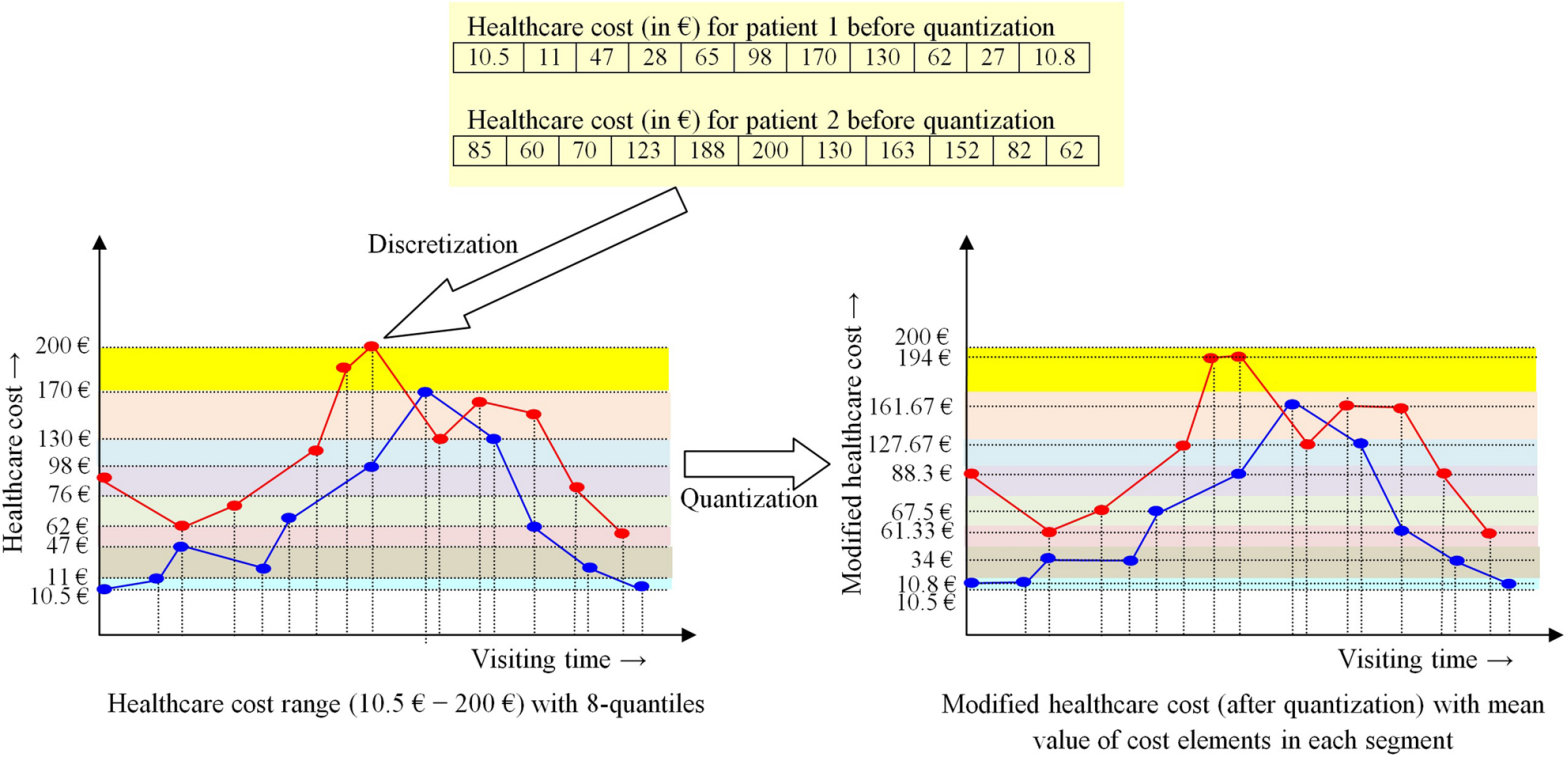
Calculation of modified healthcare cost of two patients with 8-quantiles.

The strategy is pictorially demonstrated in Fig. 1 for the healthcare cost information of two patients only with $n_s = 8$. The setting of $n_s = 8$ and the cost values used here are illustrative examples only. The healthcare cost, referred henceforth, denotes the modified cost.

### Clustering patients’ action-sequences

It is noteworthy that patients undergoing various clinical activities reveal considerable diversity of their corresponding cost information. Hence, prior to predict cost of a new action-sequence, we cluster the action-sequences of the existing patients into groups. We then consult the cost information of the specific group of patients providing the maximum similarity with the action-sequence of the new patient to predict the respective possible future cost.

Two significant issues to categorize the patients based on their action sequences include: (1) design of an appropriate distance measure to capture the similarity between action-sequences of varying length, and (2) selection of an efficient clustering algorithm to ensure that action-sequences within a group are similar to each other than those in other groups.

#### Design of distance measure

There exists plethora of literature on using *edit distance*^13^ to measure the dissimilarity of two strings of characters (or words). Given two strings $S_1$ and $S_2$ over a finite alphabet, an edit distance $ED(S_1, S_2)$ between $S_1$ and $S_2$ can be defined as the minimum cost of transforming $S_1$ to $S_2$ through a sequence of weighted edit operations. These operations primarily include insertion, deletion, and substitution of one symbol by another. Usually, the edit operations are assigned with equal weights of unity. Nevertheless, the string in this paper denotes the action-sequences.

However, there is a major limitation of using the conventional *ED* directly in the present context. The conventional *ED* compares two strings of characters (or words) only. In the present work, the components of the string (or action-sequence) is not only representing character (symbolizing a clinical activity) but an activity-cost pair. Hence, application of the conventional *ED* in the present scenario captures the difference between two action-sequences based on their respective clinical activities only, ignoring the corresponding healthcare cost information. It thus loses the cost information and the temporal relationship of the activity-cost pairs over time.

Consequently, the clusters of patients based on the conventional *ED* measures identify patients ensuring similar clinical activities only. Evidently, the accuracy of the healthcare cost prediction based on the clusters, thus formed, is reduced to great extent. It has motivated us to design an appropriate distance measure to jointly capture the dissimilarity of two clinical activities (of two different action sequences) and their respective healthcare costs.

The proposed distance measure, referred to as *treatment pattern difference* (*TPD*) is an extended version of the conventional *ED*. In case of the conventional *ED*, all possible edit operations are associated with equal cost of unity. In *TPD*, the edit costs are modified as follows to consider the healthcare cost components of two action-sequences.

Let $P_1$ and $P_2$ be two different action-sequences. The cost of insertion of a clinical activity $x_i$ (or a character) to convert $P_2$ to $P_1$ is given by

4 $$\begin{aligned} C_1 = y_i \end{aligned}$$

where $y_i$ denotes the healthcare cost of the clinical activity $x_i$ at the visiting time $t_i$ in the action-sequence $P_1$. Similarly, the cost of deleting an action $x_j$ from $P_1$ to covert it to $P_2$ is given by

5 $$\begin{aligned} C_2 = y_j \end{aligned}$$

where the symbols carry their usual meanings. If the clinical activity $x_i$ of $P_1$ is substituted with a different clinical activity $x_j$ of $P_2$, the corresponding edit cost is given by

6 $$\begin{aligned} C_3 = |y_i - y_j + \epsilon |. \end{aligned}$$

Here $\epsilon$ is a small positive constant. It is used to ensure that even when $y_i=y_j$ for $x_i \ne x_j$, at least $C_3 = \epsilon$ is used as the edit cost for substitution of $x_i$ by $x_j$.

It is noteworthy that if $x_i=x_j$, the conventional *ED* gives a zero penalty. However, there are instances of different healthcare costs for the same clinical activity of two different patients. To capture this, *TPD* uses an additional edit cost, given by

7 $$\begin{aligned} C_4 = |y_i - y_j|. \end{aligned}$$

Hence, the total edit cost to convert an action-sequence $P_1$ to another action-sequence $P_2$ is given by

8 $$\begin{aligned} TPD(P_1, P_2) = w_1\times \left( \sum _{\forall ins.} C_1 + \sum _{\forall del.} C_2 + \sum _{\forall sub.} C_3\right) + w_2\times \sum _{\forall match} C_4. \end{aligned}$$

**Figure 2:**
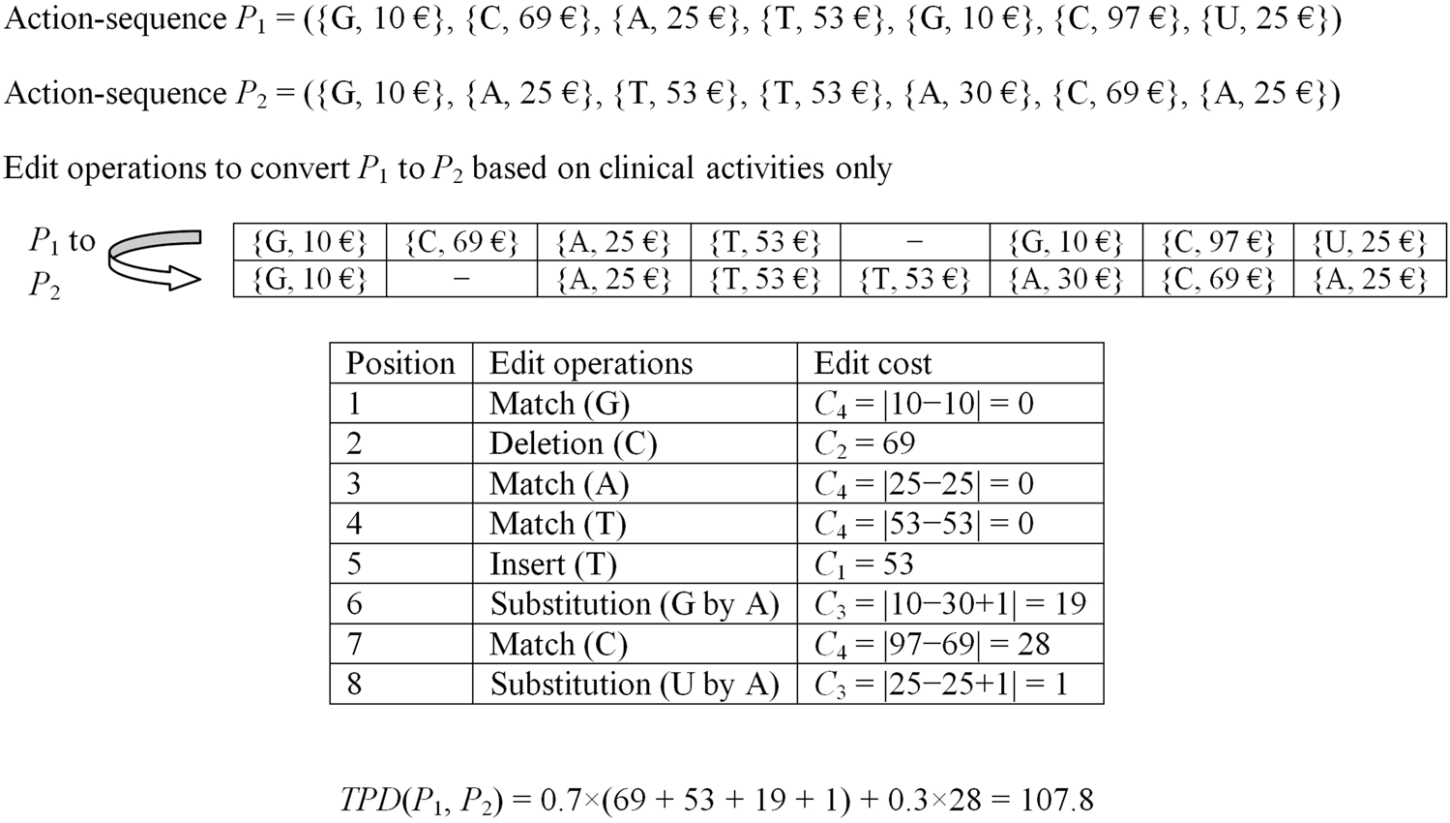
Calculation of *TPD* of two action sequences.

Here, $w_1$ and $w_2$ denote the weight for the edit operations respectively for different and similar activities. Intuitively, $w_2 < w_1$ as it corresponds to the penalty corresponding to similar activities with different healthcare cost. After a wide experimentation, we set $w_1 = 0.7$ and $w_2 = 0.3$. An example of evaluating the dissimilarity of two action-sequences based on the *TPD* measure is presented in Fig. 2.

#### Selection of clustering algorithm

The *TPD* measures of each pair of patients’ treatments in the given record are used to cluster the similar sequences in the same subgroups. The *hierarchical density-based spatial clustering of applications with noise* (hierarchical DBSCAN) algorithm^10^ is employed to identify the groups of patients’ treatments. The selection of DBSCAN in the present context is justified because of its merit of clustering similar data points (here, the action-sequences of patients) into same groups based on the density (number of nearby neighbors) without prior setting of the number of clusters. Moreover, unlike the traditional partitioning algorithms, DBSCAN can be applied for clusters of arbitrary shape, even when the data may be contaminated with noise^14^.

It is however worth mentioning that the huge economic database includes clusters of records of patients characterized at different density levels. The traditional DBSCAN algorithm with a single global density threshold often fails to effectively identify such clusters. This impasse is overcome here by using the hierarchical DBSCAN, proposed in^10^, which discovers all DBSCAN-identified clusters for an infinite range of density thresholds. Finally, it identifies a simplified hierarchical structure of significant clusters only.

### Markov chain representation of a cluster

This step is concerned with representing each cluster of patients’ action-sequences by a Markov chain^11^. The crux of such representation is founded on the underlying premise that the medical practitioners take their decision based on the previous clinical activities. Again, our cost prediction algorithm greatly relies on the recorded action-sequence of a patient.

A first order Markov chain exhibits memoryless property where the current state only depends on the previous state. Let *N* be the possible number of actions (activity-cost pairs) in the database. The Markov chain model of a group of patients, say $G_l$, is then demonstrated by a state-transition probability distribution, which is denoted as:

9 $$\begin{aligned}&M_l = [m_{i,j,l}] \qquad \text {for} \qquad i,j = 1, 2, \ldots , N \end{aligned}$$

10 $$\begin{aligned}&\text {where}\quad m_{i,j,l} = p_{l} (x_{t+1}=s_{j}|x_{t}=s_{i}) = \frac{q_{i,j,l}}{\sum _{k=1}^{N}q_{i,k,l}}. \end{aligned}$$

Here $q_{i,j,l}$ and $p_l(x_{t+1}=a_j|x_t=a_i)$ respectively denote the number of cases and the probability of transition from the current action $x_t=a_i$ to the immediate next action $x_{t+1}=a_j$ in the specific group $G_l$ of action-sequences. Evidently, it satisfies

11 $$\begin{aligned} m_{i,j,l} \ge 0 \qquad \text {and} \qquad \sum _{j=1}^{N}m_{i,k,l} = 1. \end{aligned}$$

In addition to $M_l$, we also evaluate the initial probability $p_l(a_i)$ of action $a_i$ considering all the action-sequences in the group $G_l$ for $i = 1, 2, \ldots , N$ as follows.

12 $$\begin{aligned} p_l(a_i) = \frac{s_{i,l}}{\sum _{k=1}^{n}s_{k,l}} \end{aligned}$$

Here $s_{i,l}$ denotes the number of action-sequences initiated with the action $a_i$ in $G_l$ for $i = 1, 2, \ldots , N$. This entire process is repeated for all groups identified by the hierarchical DBSCAN.

### Cost prediction of a patient’s treatment from action sequence

The aim of this step is to predict the possible total cost of a patient from the respective action-sequence. The action-sequence of the patient is formed following the principle given in “Data transformation” section. Let the ordered sequence of actions of the new patient’s treatment be denoted by $P = (a_1, a_2, \ldots , a_n)$ where the action $a_i$ represents the activity-cost pair at the visiting time instant $t_i$. The prediction of future cost based on *P* is undertaken in three phases.

#### Phase-1: cost estimation of *P* based on a specific group

We employ *k*-nearest neighbor (*k*-NN) to identify *k* action-sequences from a group, say $G_l$, that offer maximum similarity with *P* based on *TPD* measure as given in (8). First, we compute the *TPD* values between *P* and each member sequence of the group $G_l$. The member sequences are then sorted in ascending order of their *TPD* measures thus evaluated. The first *k* members are selected as the *k* nearest neighbors of *P*. Next, each of the *k* members is assigned a weight $w_{j,l}$, inversely proportional to its *TPD* measure from *P* for $j = 1, 2, \ldots , k$. Consequently, the total cost $\hat{c}_l(P)$ of the new action-sequence *P* estimated by the group $G_l$ is given by

13 $$\begin{aligned} \hat{c}_l(P) = \frac{\sum _{j=1}^{k}w_{j,l}\times c_{j,l}}{\sum _{j=1}^{k}w_{j,l}}. \end{aligned}$$

Here $c_{j,l}$ denotes the total cost incurred by the *j*-th nearest neighbor of *P* in $G_l$ for $j = 1, 2, \ldots , k$. $\hat{c}_l(P)$ is computed for all clusters of patients identified by the hierarchical DBSCAN.

#### Phase-2: Evaluation of the likelihood of *P* to patients’ groups

This step is concerned with evaluating the likelihood of *P* to each subgroup of patients based on the respective Markov chain model. The likelihood of the ordered sequence of actions $P = (a_1, a_2, \ldots , a_n)$ to a specific group $G_l$ is given by

14 $$\begin{aligned} \lambda _l(P) = p_l(a_1) \times \prod _{i=1}^{n-1}p_l(a_{i+1}| a_i). \end{aligned}$$

Here $a_1$ denotes the initial action of *P* and $a_i$ represents the action of *P* occurred at visiting time $t_i$ for $i = 1, 2, \ldots , n$. Evidently, $p_l(a_1)$ and $p_l(a_{i+1}| a_i)$ respectively symbolize the initial probability of action $a_1$ and the probability of transition from the current action $a_i$ to the immediate next action $a_{i+1}$ of *P* as described by the group $G_l$. Expression (14) is evaluated using the Markov chain model $\mathbf{M} _l$ representing the group $G_l$.

After evaluating $\lambda _l(P)$ for all groups, the normalized likelihood of *P* to each subgroup is computed using

15 $$\begin{aligned} \hat{\lambda } _l(P) = \frac{\lambda _l(P)}{\sum _{\forall k}^{} \lambda _k(P)}. \end{aligned}$$

#### Phase-3: Cost prediction based on all groups

After evaluating the estimated cost and the normalized likelihood of *P* to all groups, the total cost of *P* is finally predicted following

16 $$\begin{aligned} \bar{c}(P) = \sum _{\forall l}^{}\hat{\lambda }_l(P) \times \hat{c}_l(P). \end{aligned}$$

## Results

### Database

The study is performed on the economic data, along with the clinical activities of the patients obtained from the health administrative department of the public health care system (OSAKIDETZA) of the Basque Country, Spain. The database includes medical history of 579798 patients treated in different levels of healthcare organizations (including 1 hospital, 11 outpatients clinics and emergency care) from January 1, 2017 to December 31, 2019. The clinical data of the patients primarily consists of their clinical assistance and the respective healthcare cost information.

To validate the proposed method of cost prediction, the present work considers the pool of breast cancer patients only. The selection of breast cancer patients from the database conforms the International Statistical Classification of Diseases and Related Health Problems (10-th revision)^15^, stating that every code starting by C50 corresponds to breast cancer diagnosis. A few filtering steps are then carried out following^16^ to judiciously select the pool of patients of interest. The filtering process affirms that the selected patients have their complete treatment in the above-mentioned time period of two years. Following the medical guideline, a final set of 972 patients is identified. $70\%$ of the entire database is ultimately used as the training dataset, while the remaining as the test data. A 10-fold cross validation is employed on the training dataset for judicious selection of the value of *k* for *k*-NN.The proposed method is implemented using MATLAB 2019a.

### Identification and representation of patients’ action-sequences

**Figure 3:**
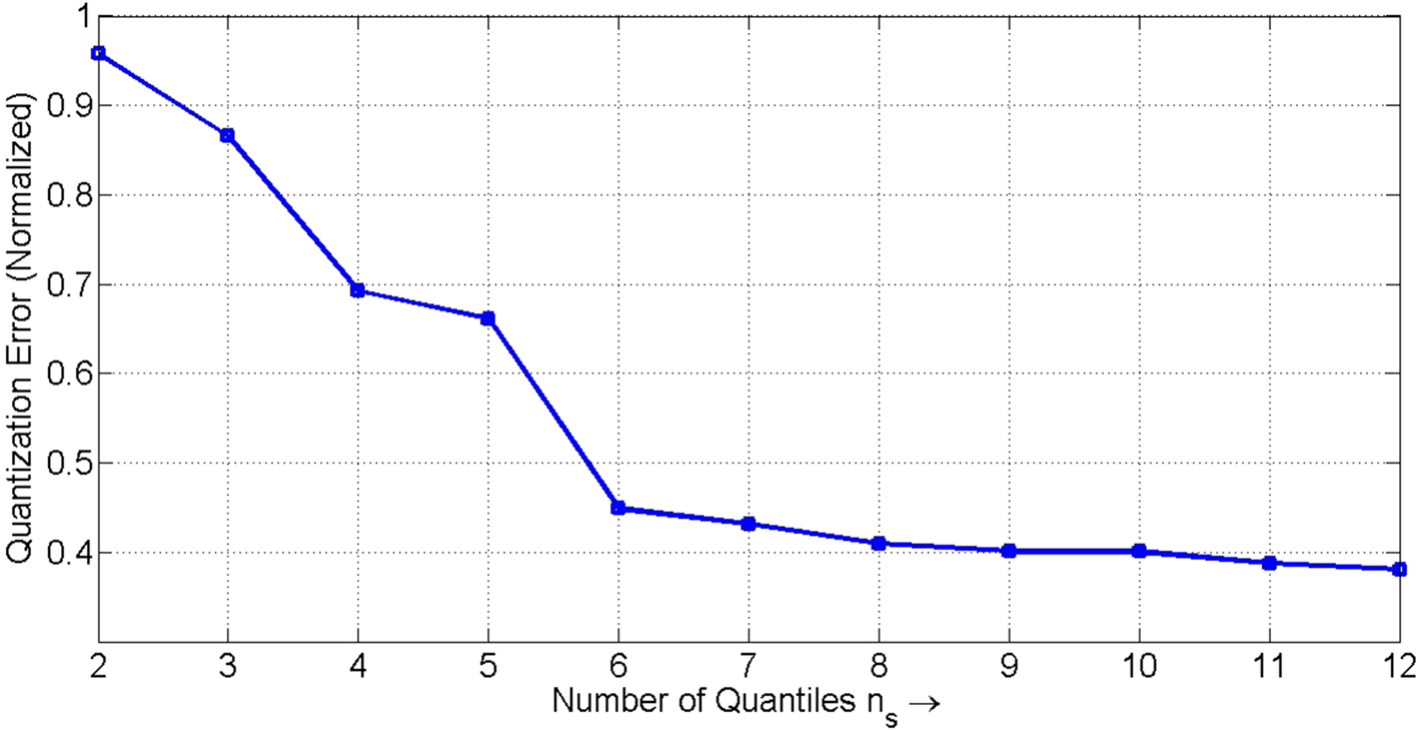
Normalized quantization error for different values of $n_s$.

The final record of the 464 patients consists of 23 unique clinical activities as described in Table 1. The healthcare cost is next discretized into $n_s$ segments. In Fig. 3, we present a plot of normalized quantization error values for different settings of the number of quantiles $n_s$, varied from 2 to 12 to check a significant improvement in performance. The normalized quantization error (*NQE*) is given by (17).

17 $$\begin{aligned} NQE= \frac{\frac{1}{N_c} \sum _{i=1}^{N_c} \vert c(i) - c_m(i) \vert }{\max _{i=1}^{N_c}c(i) - \min _{i=1}^{N_c}c(i)} \end{aligned}$$

Here *c*(*i*) and $c_m(i)$ respectively denote the true and the modified *i*-th healthcare cost (after discretization) of the database with $N_c$ cost elements for $i = 1, 2, \ldots , N_c$. Figure 3 reveals that the quantization error is reduced with an increase in the number of segments $n_s$. However, it is also observed that there is no significant change in the error for $n_s\ge 8$. We have thus fixed $n_s = 8$. It is worth mentioning that the setting of $n_s$ here is biased to the healthcare cost values of the present database. The quantization of the healthcare cost range of the present database using 8-quantiles ensures a balanced number of healthcare cost elements in each of the eight cost-segments.

**Table 1:** Description of the clinical activities.

Activity	Abbreviated form	Full form
1	ANES	Aesthesia
2	APAT	Pathological Anatomy
3	CEXT	External Consultation
4	CONS	Consultation
5	FAMB	Hospital Pharmacy Services
6	FAMR	Pharmacy
7	HCRI	Critical Care Hospitalization
8	HDIA	Day Hospital
9	HDOM	Home Hospitalization
10	HOSP	Hospitalization
11	INCO	Interconsultation
12	LABO	Laboratory
13	MNUC	Nuclear Medicine
14	OSAT	Osatek (Magnetic Resonance Service)
15	PFUN	Functional Testing
16	QUIR	Surgery Unit
17	RADI	Radiology
18	REHA	Rehabilitation
19	RTER	Radiotherapy
20	UCRI	Nursing Critical Care Unit
21	UCSI	Surgery without Hospitalization
22	UENF	Nursing Unit
23	URP	Post Anesthesia Care Unit

Next, the healthcare cost of all clinical activities of 464 patients is discretized in eight segments based on 8-quantiles of the healthcare cost range, as demonstrated in Fig. 1. Let the segments (sorted in ascending order) be denoted as *very-very-low* (*VVL*), *very-low* (*VL*), *low* (*L*), *medium-low* (*ML*), *medium-high* (*MH*), *high* (*H*), *very-high* (*VH*) and *very-very-high* (*VVH*). Eventually, there exist $22 \times 8 = 176$ actions to jointly represent a pair of clinical activity and the corresponding healthcare cost. However, a close scrutiny of the final record reveals only 63 possible pairs from the recorded medical history of the 464 patients, as reported in Table 2.

**Figure 4:**
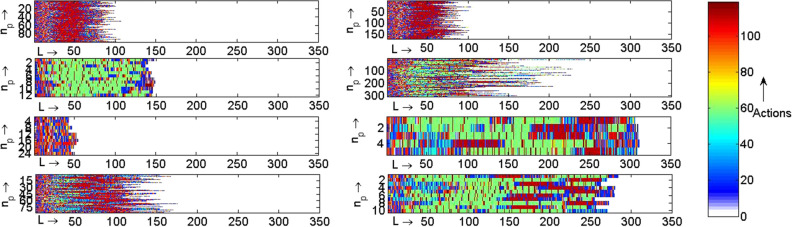
Cluster of sequences of visit records (activity-cost pairs) of patients with $\textit{n}_{{p}}$ as number of patients and *L* as the length of the sequence.

The hierarchical DBSCAN algorithm is then employed on the training dataset to cluster the sequences using *TPD* values. The algorithm results in eight clusters. The clusters thus identified are pictorially represented in Fig. 4. The descriptions of the actions of the sequences, shown in different colors, are tabulated in Table 2. Each cluster is then described by a Markov chain following “Markov chain representation of a cluster” section.

**Table 2:** Description of the clinical actions (activity-cost pairs).

Action	Activity	Cost	Action	Activity	Cost	Action	Activity	Cost
**(A)**								
1	ANES	VVL	26	FAMB	L	51	HDIA	VVH
2	ANES	VL	27	FAMB	ML	52	HDOM	VVL
3	ANES	L	28	FAMB	H	53	HDOM	VL
4	ANES	ML	29	FAMB	VH	54	HDOM	L
5	ANES	MH	30	FAMB	VVH	55	HDOM	ML
6	ANES	H	31	FAMR	VVL	56	HDOM	H
7	ANES	VH	32	FAMR	VL	57	HDOM	VH
8	ANES	VVH	33	FAMR	L	58	HDOM	VVH
9	APAT	VVL	34	FAMR	ML	59	HOSP	VVL
10	APAT	L	35	FAMR	MH	60	HOSP	VL
11	APAT	VH	36	FAMR	H	61	HOSP	L
12	APAT	VVH	37	FAMR	VH	62	HOSP	ML
13	CEXT	VL	38	FAMR	VVH	63	HOSP	MH
14	CEXT	L	39	HCRI	VVL	64	HOSP	H
15	CEXT	ML	40	HCRI	VL	65	HOSP	VH
16	CEXT	H	41	HCRI	L	66	HOSP	VVH
17	CONS	VVL	42	HCRI	VH	67	INCO	L
18	CONS	L	43	HCRI	VVH	68	INCO	ML
19	CONS	ML	44	HDIA	VVL	69	INCO	MH
20	CONS	MH	45	HDIA	VL	70	INCO	VH
21	CONS	H	46	HDIA	L	71	INCO	VVH
22	CONS	VH	47	HDIA	ML	72	LABO	L
23	CONS	VVH	48	HDIA	MH	73	LABO	MH
24	FAMB	VVL	49	HDIA	H	74	LABO	VH
25	FAMB	VL	50	HDIA	VH	75	LABO	VVH
**(B)**								
76	MNUC	L	91	QUIR	MH	106	REHA	ML
77	MNUC	ML	92	QUIR	H	107	REHA	MH
78	MNUC	H	93	QUIR	VH	108	RTER	VVL
79	MNUC	VH	94	QUIR	VVH	109	RTER	MH
80	MNUC	VVH	95	RADI	VVL	110	RTER	H
81	OSAT	L	96	RADI	VL	111	RTER	VH
82	OSAT	H	97	RADI	L	112	RTER	VVH
83	OSAT	VH	98	RADI	ML	113	UCRI	VVH
84	OSAT	VVH	99	RADI	MH	114	UCSI	VH
85	PFUN	VVL	100	RADI	H	115	UENF	MH
86	PFUN	VL	101	RADI	VH	116	UENF	H
87	PFUN	L	102	RADI	VVH	117	UENF	VH
88	QUIR	VL	103	REHA	VVL	118	UENF	VVH
89	QUIR	L	104	REHA	VL	119	URP	ML
90	QUIR	ML	105	REHA	L

### Performance evaluation of proposed healthcare cost prediction method

#### Performance metric

The performance of the proposed cost prediction algorithm is evaluated with respect to *mean absolute percentage error* (*MAPE*) with a lower error indicating a better performance.

18 $$\begin{aligned} MAPE= \frac{\frac{1}{N_t} \sum _{i=1}^{N_t} \vert c(P_i) - \bar{c}(P_i) \vert }{\frac{1}{N_t} \sum _{i=1}^{N_t}c(P_i)} \times 100. \end{aligned}$$

Here $c(P_i)$ and $\bar{c}(P_i)$ (evaluated using (16)) respectively represent the true and the predicted cost of the *i*-th patient’s treatment $P_i$ in the validation or the test dataset with $N_t$ records for $i = 1, 2, \ldots , N_t$.

#### Validation of earliness prediction and selection of *k* of *k*-NN

The capability of the proposed algorithm to predict the possible total healthcare cost of patients is verified by varying the length of sequence of the recorded treatments of the patients from 20 to 100%. The appropriate selection of *k* (of *k*-NN) for the optimal performance is undertaken using 10-fold cross validation on the training dataset. The *MAPE* values (averaged over 10 folds of the training data) for different settings of *k* and percentage of length of sequence of the recorded treatments of the patients are tabulated in Table 3. Table 3 reveals that the longer the length of the sequence, the better is the prediction accuracy with smaller *MAPE* measures, irrespective of the setting of *k*. The optimal performance of the method is obtained for $k = 3$ with the entire sequence information. It is also noted that an *MAPE* smaller than 6% is obtained even when 50% of a visit sequence is utilized. It proves the effectiveness of the proposed method for an early prediction of the healthcare cost.

**Table 3:** *MAPE* values (with training data during 10-fold cross validation) for different values of *k* and length of action sequence (in percentage).

*k*	Length of action sequence (in percentage)								
	$20\%$	$30\%$	$40\%$	$50\%$	$60\%$	$70\%$	$80\%$	$90\%$	$100\%$
1	9.25	7.43	6.01	6.50	5.85	5.53	4.68	4.15	3.76
2	8.83	8.08	6.95	6.54	6.04	5.80	5.15	4.10	3.65
3	9.41	8.87	5.89	5.39	4.98	4.62	4.38	4.07	3.49
4	9.36	7.82	6.04	5.47	4.86	4.65	3.94	3.77	3.63
5	8.90	7.14	5.69	5.13	4.84	4.49	4.24	3.77	4.03
6	9.01	7.35	5.76	5.32	5.17	5.15	4.51	4.26	4.33
7	9.29	7.58	5.77	5.27	5.58	4.97	4.62	4.46	4.35
8	9.13	7.39	5.72	5.64	5.18	5.28	4.46	4.13	4.21
9	9.57	7.66	5.92	5.42	5.57	5.14	4.64	4.11	4.08
10	9.68	8.51	6.27	6.26	6.11	5.93	5.22	4.60	4.41

**Figure 5:**
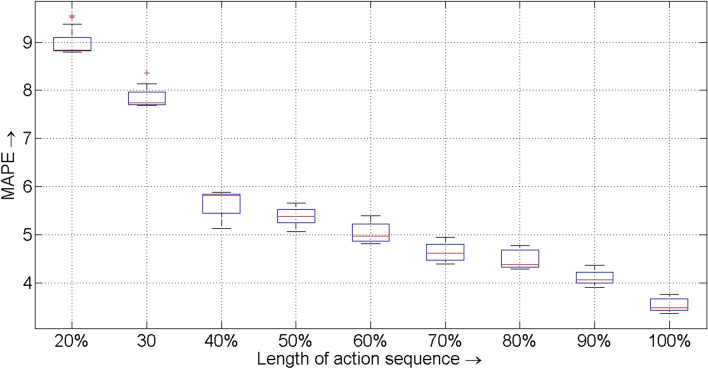
Boxplot of *MAPE* values obtained by the proposed method for different length of action sequence over 20 experimental runs (each with 10 fold cross validation of the training data).

Next to check the variability of the *MAPE* measures obtained by the proposed method with $k = 3$, 20 experimental runs are undertaken with 10-fold cross validation of the training data. The samples of each of the 10-folds of 20 runs are randomized. The results are summarized in Fig. 5. Figure 5 reveals detection of outliers when the length of the action sequences (i.e., squence of recorded treatments) is considerably small. The mean and standard deviation of the *MAPE* values obtained by the proposed method over 20 experimental runs, each with 10 folds, are reported in Table 4.

**Table 4:** Mean and standard deviation (STD) of *MAPE* values obtained by the proposed method over 20 experimental runs (each with 10-fold cross validation of training data) for different length of action sequence (in percentage *per*).

*per*	$20\%$	$30\%$	$40\%$	$50\%$	$60\%$	$70\%$	$80\%$	$90\%$	$100\%$
Mean	9.01	7.86	5.65	5.39	5.05	4.64	4.49	4.12	3.54
STD	0.28	0.19	0.27	0.17	0.23	0.21	0.18	0.15	0.13

#### Comparative performance analysis

The next experiment aims at comparative performance analysis of our proposed algorithm. Three state-of-the-art techniques are considered in the comparative framework, including *gradient boosting* (GB)^17^, *artificial neural net* (ANN)^18^ and *elastic net* (EN)^19^. These existing methods have utilized the healthcare cost data only to predict the future cost^2^. The hyperparameters of the competitive algorithms are tuned using the grid search method and the 10-fold cross validation of the training data. The tuned hyperparameters are reported in Table 5.

**Table 5:** Tuned hyperparameters of competitive methods.

Algorithms	Hyperparameters	Range considered	Selected value
GB	No. of estimators	[2, 3, ..., 60]	45
	Learning rate	[0, 0.05, ..., 1]	0.05
	Subsample	[0.2, 0.25, ..., 0.9]	0.75
	Max tree depth	[2, 3, ..., 20]	8
ANN	No. of hidden layers	[1, 2, ..., 5]	1
	Learning rate	[0, 0.05, ..., 1]	0.1
	Momentum factor	[0.1, 0.2, ..., 1]	0.5
	Activation function	[sigmoid, tanh, ReLU]	sigmoid
EN	Penalty weight	[$10^{-5}$, ..., 10]	0.2
	Mixing parameter	[0, 0.01, ..., 1]	0.62

The comparative analysis of performance of the competitors is undertaken next. To ensure fair comparison of all contenders, each algorithm is evaluated on the same 10-fold cross validation split of the data and the same random number seed is used to split the data in each case. The *MAPE* measures (averaged over 10 folds) obtained by all four contender algorithms using training data are tabulated in Table 6. Table 6 reveals that the proposed algorithm outperforms its contenders by achieving the minimum *MAPE* measure in most of the cases. GB outperforms the proposed method in two cases, where the lengths of action sequence are 20% and 30%.

**Table 6:** Mean *MAPE* values (with training data during 10-fold cross validation with same random number seed) for different competitive methods for different length of action sequence (in percentage).

Algo.	Length of action sequence (in percentage)								
	$20\%$	$30\%$	$40\%$	$50\%$	$60\%$	$70\%$	$80\%$	$90\%$	$100\%$
Prop. method	9.41	8.87	5.89	5.39	4.98	4.62	4.38	4.07	3.49
GB	8.74	8.37	8.22	8.17	7.74	7.67	5.38	4.79	4.30
ANN	11.95	10.74	9.54	8.11	8.02	7.59	7.43	6.74	6.41
EN	10.56	10.37	10.06	9.57	8.91	8.39	7.32	7.22	6.79

The results of Table 6 are further used to carry out the hypothesis test to verify the statistical significance of the difference in performance of the proposed algorithm and each of its three contenders. Assuming no specific distribution of the population of *MAPE* values (obtained after 10-fold cross validation for each algorithm), the Friedman non-parametric test^20^ is undertaken on the mean values of *MAPE* metric obtained by the contender algorithms (over 10-fold cross validation) with a level of significance $\alpha$ = 0.05. The Friedman ranks obtained by the contender algorithms based on the results given in Table 6 are reported in Table 7. The results reported in Table 7 also designate the proposed method as the best algorithm. The test considers the null hypothesis that there is no significant difference between the performances (based on the mean *MAPE* measures) of the competitive algorithms. Table 8 however reveals that the resulting Friedman test statistic value = 14.133 exceeds the respective critical value of 7.815 following $\chi _F^2$ distribution with 3 degrees of freedom at $\alpha$ = 0.05. It substantiates statistically significant difference between the *MAPE* measures obtained by the proposed algorithm and each of its contenders.

**Table 7:** Friedman ranks obtained by contender algorithms.

Algo.	Friedman rank
Prop. method	1.222
GB	2.000
ANN	3.111
EN	3.667

**Table 8:** Statistical values obtained by Friedman test based on Friedman ranks given in Table 7.

Non-parametric test	Friedman
Statistic value	14.133
Degree of freedom	3
Critical value	7.815
Comment on null hypothesis	Reject

**Figure 6:**
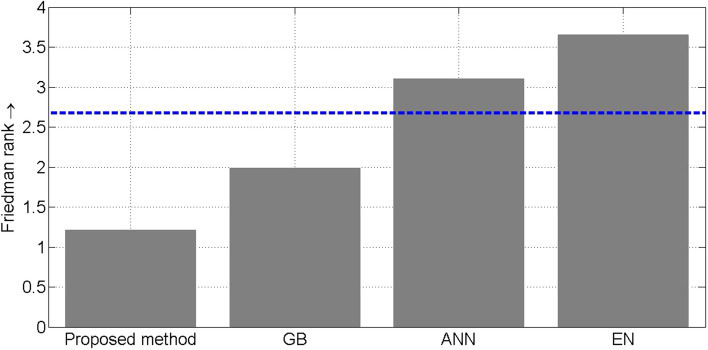
*MAPE* obtained by different competitive algorithms for different length of action sequence (test data).

The results in Table 7 highlights the proposed method as the best algorithm, so the Bonferroni–Dunn post-hoc analysis^20, 21^ is performed with the proposed method as the control method. For the Bonferroni–Dunn test, a critical difference value is calculated which for these data (represented by Table 7) comes as 1.457. A significant difference between the performances of the control algorithm and its contender is inferred if their corresponding Friedman ranks differ at least by a critical difference. Pictorially, it is shown in Fig. 6. It is evident that the performances of ANN and EN in the present context are significantly inferior to the proposed method.

Finally, following the inferences from the non-parametric statistical test, the Hochberg multiple comparison test is further undertaken^21^ with the proposed method (achieving the best Friedman rank) as the control algorithm. The adjusted *p*-values are reported in Table 9. It is evident from Table 9 that the test infers that there is no statistically significant difference between the performances of the proposed method and GB with the respective adjusted *p*-value exceeding $\alpha = 0.05$^22^. However, the null hypothesis is rejected for the remaining cases of comparing the proposed method with its competitor algorithms with an adjusted *p*-value smaller than $\alpha = 0.05$.

**Table 9:** Adjusted *p*-values using Hochberg multiple comparison test.

Comp. Algo.	*z*-score	Unadjusted *p*-value	Adjusted *p*-value
GB	1.278	$2.012\times 10^{-1}$	$2.012\times 10^{-1}$
ANN	3.104	$1.911\times 10^{-3}$	$3.822\times 10^{-3}$
EN	4.0167	$5.900\times 10^{-5}$	$1.770\times 10^{-4}$

**Figure 7:**
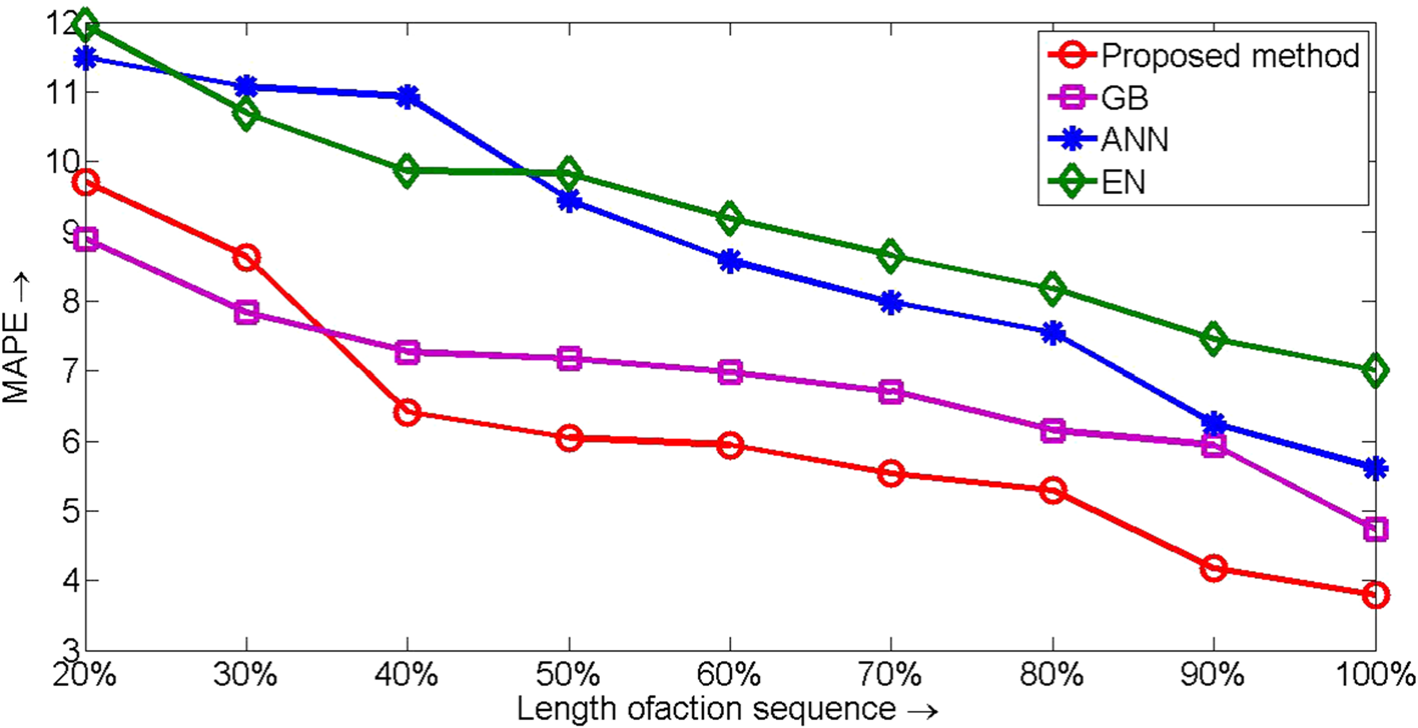
*MAPE* obtained by different competitive algorithms for different length of action sequence (test data).

Table 10 reports the *MAPE* measures for the same competitors for the test data. The reported results are pictorially presented in Fig. 7. The reported results substantiate that our proposed method overcomes its contenders with GB acquiring the second rank. It in turn validates the efficiency of jointly considering the clinical activity and the associated cost data for the healthcare cost prediction.

**Table 10:** *MAPE* values (with test data) for different competitive methods for different length of action sequence (in percentage).

Algo.	Length of action sequence (in percentage)								
	20%	$30\%$	$40\%$	$50\%$	$60\%$	$70\%$	$80\%$	$90\%$	$100\%$
Prop. method	9.70	8.63	6.41	6.05	5.94	5.53	5.29	4.17	3.79
GB	8.89	7.84	7.27	7.17	6.98	6.71	6.15	5.94	4.73
ANN	11.49	111.07	10.93	9.45	8.58	7.98	7.55	6.95	6.61
EN	11.95	10.69	9.87	9.83	9.18	8.65	8.18	7.45	7.01

## Conclusion

The paper presents a novel method to predict healthcare cost of breast cancer patients as early and accurately as possible. The early prediction capability of the proposed method is used for identifying patients at risk of becoming high-cost healthcare users, before incurring substantial avoidable costs. The merit of the paper lies in the following counts. First, it considers the clinical activity and the associated healthcare cost data jointly to model the treatment of a patient. Second, it recommends a novel distance measure to capture the dissimilarity of two treatment patterns, encompassing both clinical activities and healthcare cost information. Third, it employs the hierarchical DBSCAN to categorize patients into different clusters with an aim to effectively identify the high-need and/or high-cost patients. Fourth, each cluster of patients is depicted by a Markov chain model to mathematically represent the treatment patterns. Finally, the Markov chain models of all the clusters are used to predict the possible future (total) cost of a patient’s treatment. The performance of the proposed algorithm is compared for different length of sequence of the recorded treatments of patients. The experimental results reveal that the method achieves an *MAPE* value, as small as 6% even with half of the clinical records of a patient. Experiments undertaken also substantiate the superiority of the proposed algorithm to three state-of-the-art techniques which utilize only the healthcare cost data of the patients for prediction.

As a continuation of the present work, we first plan to test our method on different databases from different healthcare organizations for patients suffering from different diseases. More experiments on different databases could help to take a deeper dive into the data and explore ways to obtain more solid evidence on the performance of the proposed method, irrespective of databases. Second, we may consider the socio-demographic information of the patients along with the clinical actions with an aim to be utilize their joint explanatory power to understand the root causes of patient’ costs. Third, we have not exploited time feature in the present work. Intuitively, inclusion of time feature may effectively capture the differences of treatment patterns of patients and thus may enhance the prediction performance of the proposed method. Finally, appropriate stratagem needs to be developed to effectively balance the trade-off between the accuracy and earliness of the healthcare cost prediction.
